# p38 MAPK signaling in chronic obstructive pulmonary disease pathogenesis and inhibitor therapeutics

**DOI:** 10.1186/s12964-023-01337-4

**Published:** 2023-11-02

**Authors:** Ali Ahmadi, Sajjad Ahrari, Jafar Salimian, Zahra Salehi, Mehrdad Karimi, Alireza Emamvirdizadeh, Sadegh Azimzadeh Jamalkandi, Mostafa Ghanei

**Affiliations:** 1 Molecular Biology Research Center, Systems Biology and Poisonings Institute, Baqiyatallah University of Medical Sciences, Tehran, Iran; 2 Department of Biochemistry and Molecular Medicine, Institute for Research in Immunology and Cancer (IRIC), Université de Montréal, Montréal, QC Canada; 3 Applied Virology Research Center, Baqiyatallah University of Medical Sciences, Tehran, Iran; 4 Hematology-Oncology and Stem Cell Transplantation Research Center, Tehran University of Medical Sciences, Tehran, Iran; 5 Department of Traditional Medicine, School of Persian Medicine, Tehran University of Medical Sciences, Tehran, Iran; 6 Department of Molecular Genetics, Faculty of Bio Sciences, Tehran North Branch, Islamic Azad University, Tehran, Iran; 7 Chemical Injuries Research Center, Systems Biology and Poisonings Institute, Baqiyatallah University of Medical Sciences, Tehran, Iran

**Keywords:** Chronic Obstructive Pulmonary Disease, COPD, COPD pharmacotherapy, p38 MAPK inhibitors, p38 MAPK signaling, Systematic review

## Abstract

**Background:**

Chronic obstructive pulmonary disease (COPD) is characterized by persistent respiratory symptoms and airflow limitation due to airway and/or alveolar remodeling. Although the abnormalities are primarily prompted by chronic exposure to inhaled irritants, maladjusted and self-reinforcing immune responses are significant contributors to the development and progression of the disease. The p38 isoforms are regarded as pivotal hub proteins that regulate immune and inflammatory responses in both healthy and disease states. As a result, their inhibition has been the subject of numerous recent studies exploring their therapeutic potential in COPD.

**Main body:**

We performed a systematic search based on the PRISMA guidelines to find relevant studies about P38 signaling in COPD patients. We searched the PubMed and Google Scholar databases and used “P38” AND “COPD” Mesh Terms. We applied the following inclusion criteria: (1) human, animal, ex vivo and in vitro studies; (2) original research articles; (3) published in English; and (4) focused on P38 signaling in COPD pathogenesis, progression, or treatment. We screened the titles and abstracts of the retrieved studies and assessed the full texts of the eligible studies for quality and relevance. We extracted the following data from each study: authors, year, country, sample size, study design, cell type, intervention, outcome, and main findings. We classified the studies according to the role of different cells and treatments in P38 signaling in COPD.

**Conclusion:**

While targeting p38 MAPK has demonstrated some therapeutic potential in COPD, its efficacy is limited. Nevertheless, combining p38 MAPK inhibitors with other anti-inflammatory steroids appears to be a promising treatment choice. Clinical trials testing various p38 MAPK inhibitors have produced mixed results, with some showing improvement in lung function and reduction in exacerbations in COPD patients. Despite these mixed results, research on p38 MAPK inhibitors is still a major area of study to develop new and more effective therapies for COPD. As our understanding of COPD evolves, we may gain a better understanding of how to utilize p38 MAPK inhibitors to treat this disease.

Video Abstract

**Supplementary Information:**

The online version contains supplementary material available at 10.1186/s12964-023-01337-4.

## Abstract (plain language summary)

We wanted to determine what studies have been done on how a protein called p38 affects a lung disease called COPD. COPD is a condition that makes it hard to breathe and can cause coughing, wheezing, and chest infections. p38 is a protein that helps cells to respond to stress and inflammation, but it may also play a role in causing or worsening COPD. We searched two main online databases for studies that met our criteria. We looked for studies that involved humans, studies that used animals or cells in the lab, studies that reported new findings, studies that were written in English, and studies that focused on p38 and COPD. We did not include studies that were reviews, summaries, opinions, or letters or studies that were not related to p38 or COPD. We found 361 studies that matched our criteria. We read the titles and summaries of these studies and checked the full texts for quality and relevance. We collected information from each study, such as who did it, when and where it was done, how many people were involved, what type of cells were studied, what treatment was given, what outcome was measured, and what the main results were. We grouped the studies based on the type of cells and type of treatment they studied. We found that different types of cells (such as lung cells, immune cells, and blood cells) and different types of treatment can affect how p38 works in COPD.

**Supplementary Information:**

The online version contains supplementary material available at 10.1186/s12964-023-01337-4.

## Background

According to the Global Initiative for Chronic Obstructive Lung Disease (GOLD) description, “*Chronic obstructive pulmonary disease (COPD) is a heterogeneous lung condition characterized by chronic respiratory symptoms (dyspnea, cough, sputum production, exacerbations) due to abnormalities of the airways (bronchitis, bronchiolitis) and/or alveoli (emphysema) that cause persistent, often progressive airflow obstruction.*” [1]. COPD is among the top third causes of death, according to the World Health Organization (WHO) [2]. The disease is characterized by small airway complications such as obstructive bronchiolitis, fibrosis, airway remodeling, and emphysema [3]. The clinical manifestations of COPD vary among different patients [4], and an accelerated decline in lung function is observed in approximately half of patients [5]. This suggests the critical impact of personal distinctive inflammatory signatures and genetic/epigenetic makeup on disease severity [6]. Currently, there is no established cure for COPD, and the current medical interventions mostly include alleviating disease complications with bronchodilators such as inhaled long-acting β2-agonists (LABAs) and long-acting muscarinic antagonists (LAMAs) [7].

In COPD patients, there is impaired crosstalk between lung circulatory and resident immune cells, resulting in the increased recruitment of neutrophils, macrophages, and T- and B-lymphocytes inside the airway lumen of COPD patients. Inhaled irritants provoke surface macrophages and airway epithelial cells to release chemokines, which recruit inflammatory cells to the lungs. However, this inflammation persists even after quitting smoking, possibly due to the self-sustaining feedback loop that hinders the alleviation of the inflammatory responses [8]. This unbalanced and dysregulated regulation leads to chronic inflammation, fibrosis, cell death, and extracellular matrix (ECM) destruction (i.e., airway remodeling) in the lungs of COPD patients.

Signal transduction regulatory mechanisms, including posttranslational phosphorylation of proteins, play a crucial role in COPD pathogenesis. Mitogen-activated protein kinases (MAPKs) are a diverse group of these kinases involved in many signal transduction pathways [9]. There are four well-characterized subfamilies of this group in mammalian cells, including ERK1/2 (Extracellular signal-regulated kinase 1 and 2), ERK5, JNKs (c-Jun N-terminal kinases), and p38s (Protein 38) [10]. As shown in earlier studies, p38 MAPKs are triggered via inflammatory mediators and oxidative stress, which activate many diverse downstream signaling pathways in different cells. External factors such as viral and bacterial infections and internal factors are involved in regulating the p38 MAPK signaling pathway [11].

However, given the role of chronic inflammation in COPD pathogenesis, the development of anti-inflammatory treatments for COPD has gained more attention in recent years. In this review, we conducted a comprehensive search to evaluate the significance of the p38 signaling pathway in the pathogenesis of COPD and the efficacy of available p38 inhibitors for COPD treatment.

## Our approach and method

In July 2023, we conducted a comprehensive electronic search of two prominent databases, PubMed and Google Scholar, to identify studies published in English that explored the relationship between COPD and p38 with no temporal restrictions. We searched all fields of the articles using predefined search terms “COPD” AND “p38 using EndNote online search and utilized truncation to capture different variations of the terms such as “chronic obstructive pulmonary disease”, “emphysema”, and “AECOPD”. To ensure the accuracy of our search strategy, we cross-checked the truncation terms with available MeSH terms.

To ensure the quality of our review, we conducted a meticulous screening of the titles and abstracts of the studies retrieved from our search. We selected only those studies that investigated the role of p38 MAPK signaling in COPD and its association with various cells, treatments, and clinical trials. We excluded studies that did not meet the research question’s criteria, such as those that examined the role of p38 MAPK signaling in other respiratory diseases and review articles, to ensure the relevance of the included studies in our review.

**Fig. 1:**
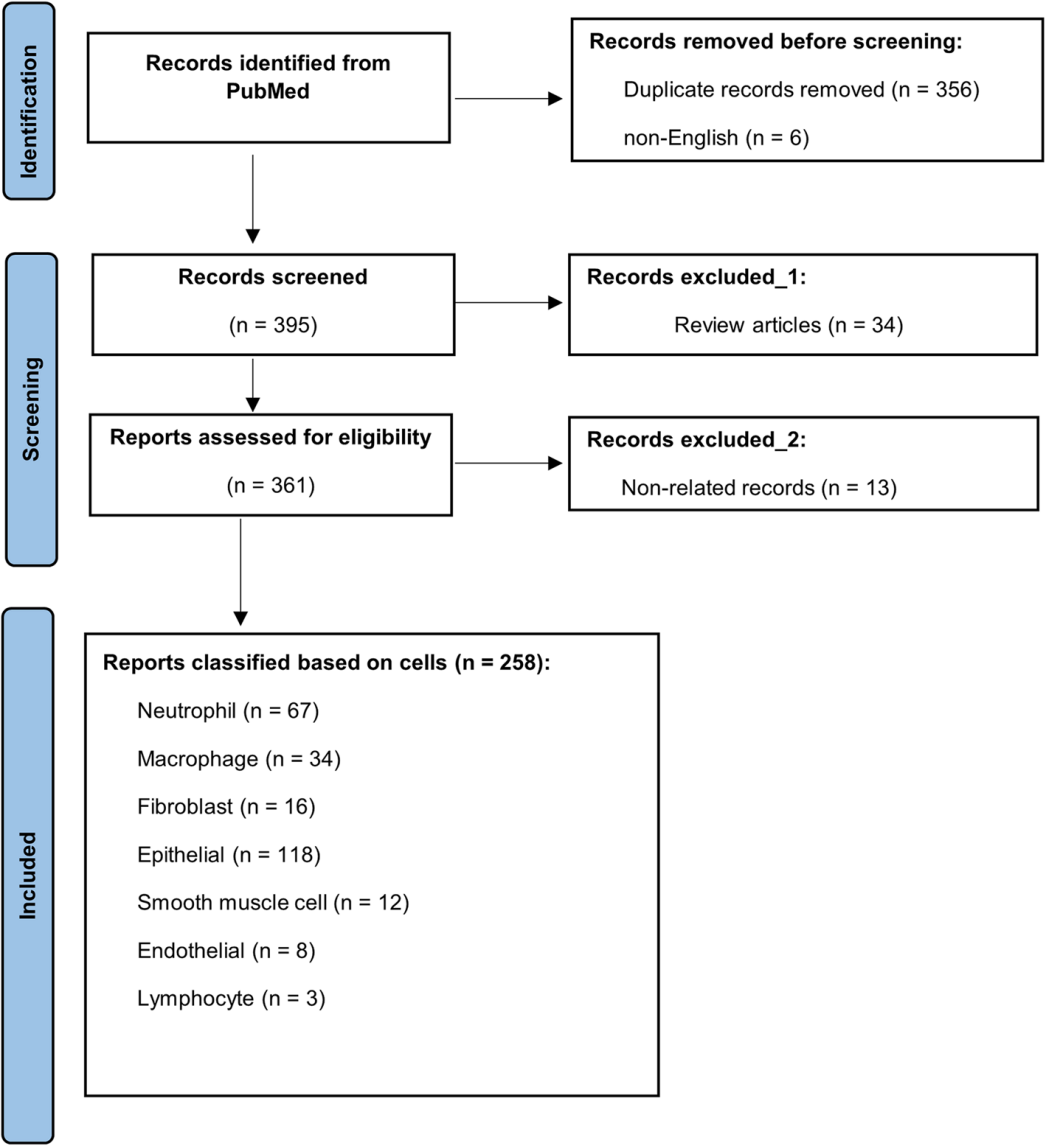
Flow Diagram Illustrating the Selection Process of Studies for Inclusion in the PRISMA Systematic Review

To identify more relevant studies, we expanded our search to include additional search terms, such as “epithelial”, “macrophage”, “fibroblast”, “neutrophil”, “smooth muscle”, “lymphocyte”, and “endothelial”, which we categorized separately. We also categorized treatments and interventions in in vivo (human and animal studies), in vitro, and ex vivo studies and subcategorized herbal interventions. After obtaining the full text of the selected studies, we conducted a comprehensive review to extract relevant data and assess evidence quality, including study design, sample size, patient characteristics, interventions, outcomes, statistical methods and findings, biological samples, organism, and key findings. We synthesized the selected studies’ findings in a narrative review and grouped them by study design, patient characteristics, and measured outcomes. The systematic search strategy is illustrated following the PRISMA standard in Fig. 1 [12].

## p38 signaling pathway

The p38 signaling pathway is a well-characterized intracellular cascade that plays a crucial role in regulating cellular responses to diverse extracellular stimuli, such as inflammatory cytokines, oxidative stress, osmotic shock, ultraviolet radiation, and growth factors. The primary mediator of the p38 pathway is a member of the mitogen-activated protein kinase (MAPK) family, which is a serine/threonine kinase family that phosphorylates a vast array of substrates in the cytoplasm and nucleus. The p38 signaling pathway is activated through a series of phosphorylation events that culminate in the activation of downstream effectors, including transcription factors, kinases, and phosphatases. This pathway is involved in numerous cellular processes, including inflammation, stress responses, cell cycle regulation, and differentiation, which underscores its importance in maintaining cellular homeostasis. Moreover, dysregulation of the p38 signaling pathway has been implicated in the pathogenesis of various diseases, such as cancer, autoimmune disorders, and neurodegenerative diseases, highlighting the need to gain a comprehensive understanding of the molecular mechanisms underlying p38 signaling.

### Positive regulation of p38 signaling

p38 MAP kinases are activated by a three-tiered mechanism, where they phosphorylate p38 on the Thr180 and Tyr182 residues. The activation of p38 is initiated by diverse types of cellular receptors, such as cytokine receptors, growth factor receptors, G protein-coupled receptors, and pattern recognition receptors [13], which involve upstream kinases such as MKK3, MKK4, MKK6, and MKK7 [13, 14]. For instance, TNFα and IL-1 activate p38 via the NF-κB pathway, insulin activates p38 using the PI3K/Akt pathway, and LPS activates p38 through the TLR4/MyD88 pathway [13]. Therefore, the cellular receptors involved in the activation of p38 may vary depending on the cell type and stimulus.

There are molecules, such as TNF-α, IL-1β, IL-6, and MCP-1, mediate immune and inflammatory responses. Cytokines can activate p38 through different receptors and signaling pathways, such as TNFR1-TRAF2-TAB1-TAK1-MKK3/6, IL-1R-TIRAP-MAL-TRAF6-TAK1-MKK3/6, IL-6R-JAK2-TYK2-MKK3/6, and CCR2-MCPIP-MKK3/6 [15, 16]. Cytokines are increased in the airways and circulation of COPD patients and contribute to chronic inflammation and tissue damage. The proinflammatory cytokines interleukin-1 beta (IL-1β) and tumor necrosis factor-alpha (TNF-α) have been shown to activate p38 signaling and promote inflammation in COPD patients [17]. Additionally, oxidative stress, which is increased in COPD due to the reduced antioxidant capacity of the lungs, can activate p38 signaling and promote inflammation [18]. Additionally, UV radiation activates the p38 pathway by causing DNA damage via the upstream kinase MKK3 [19]. Likewise, heat shock and osmotic stress induce the activation of the p38 pathway through the upstream kinases MKK6 and MKK3, respectively [20].

Oxidative stress occurs when the production of reactive oxygen species (ROS) exceeds the antioxidant capacity of the cells. ROS are involved in the activation of p38 and other stress kinases, as well as redox-sensitive transcription factors, such as NF-κB and AP-1, which regulate inflammation and tissue remodeling in COPD. Oxidative stress can activate p38 through various upstream kinases, such as ASK1, TAK1, MLK3, and MEKK1 [16]. Oxidative stress is induced by cigarette smoke and other environmental pollutants that are risk factors for COPD [21].

Moreover, there are components of the outer membrane of gram-negative bacteria that can trigger innate immune responses. LPS can activate p38 through Toll-like receptor 4 (TLR4) and its adaptor proteins, such as MyD88, TRIF, TRAM, and TRAF6, which can recruit TAK1-MKK3/6 to the receptor complex [16]. LPS can also induce oxidative stress and cytokine production that can further activate p38. LPS is present in the airways of COPD patients due to bacterial colonization or infection and can exacerbate inflammation and airflow limitation [21].

### Negative regulation of p38 signaling

The upstream regulation of p38 and other MAPKs is a crucial aspect of MAPK signaling. Phosphatases, such as protein phosphatase 2A/C (PP2A/C), wild-type p53-induced phosphatase 1 (Wip1), MAP kinase phosphatases (MKPs), and dual-specificity phosphatases (DUSPs), naturally regulate the activity of p38 and other MAPKs by dephosphorylating them and leading to their inactivation [22]. MKPs function as negative feedback regulators of MAPK signaling and can modulate inflammation, apoptosis, and oxidative stress in lung cells in response to various stimuli, such as cytokines, growth factors, and oxidative stress [23].

Conversely, protein kinases can phosphorylate and activate or inhibit p38 and other MAPKs. MAP kinase-interacting serine/threonine protein kinases (MNKs) are examples of protein kinases that inhibit p38 activation by phosphorylating and inhibiting MKK3, which is an upstream activator of p38. MNKs can also regulate the translation of several proteins involved in inflammation and fibrosis, such as TNF-α, IL-6, and collagen [15]. Additionally, MAP3Ks, which respond to different types of cellular stress, such as oxidative stress, DNA damage, cytokines, and TLR agonists, regulate p38 signaling. Apoptosis signal-regulating kinase 1 (ASK1, also known as MAP3K5), TGF-β-activated kinase 1 (TAK1, also known as MAP3K7), mixed lineage kinase 3 (MLK3, also known as MAP3K11), and mitogen-activated protein kinase kinase kinase 1 (MAP3K1, also known as MEKK1) are some examples of MAP3Ks that regulate p38 signaling [24].

Physiologically, p38 can be inhibited either directly or indirectly by anti-inflammatory agents, antioxidants, and anticancer drugs. Antioxidants act by scavenging reactive oxygen species (ROS) or by preventing their formation. ROS are involved in the activation of p38 and other stress kinases and redox-sensitive transcription factors, such as NF-κB and AP-1, which regulate inflammation and tissue remodeling in COPD. Antioxidants can attenuate p38-mediated inflammatory responses in lung cells [13]. The phosphodiesterase-4 (PDE4) inhibitor roflumilast inhibits p38 activation and reduces inflammation in COPD patients [15]. It is crucial to achieve a comprehensive understanding of the negative regulators of p38 signaling in COPD to develop innovative therapeutic strategies for COPD and identify potential therapeutic targets.

### The versatility of the p38 MAPK family

The p38 MAPK family consists of four isoforms: p38α, p38β, p38γ, and p38δ. These isoforms share a high degree of amino acid sequence homology and are activated by a variety of extracellular stimuli, including inflammatory cytokines, stress, and growth factors. p38α is the most widely studied isoform of p38 and is ubiquitously expressed in mammalian tissues. It plays a critical role in regulating cellular responses to stress and inflammation, including cell cycle progression, apoptosis, and immune responses [20].

**p38β** is closely related to p38α, sharing 75% sequence identity. It is expressed at lower levels than p38α and is primarily found in the heart and skeletal muscle, where it has been shown to play a role in regulating cardiac hypertrophy and myogenesis [25]. **p38γ and p38δ** are more recently found isoforms of p38 that have distinct tissue distributions and functions. p38γ is primarily expressed in the brain and has been implicated in regulating synaptic plasticity and memory formation. p38δ, on the other hand, is highly expressed in adipose tissue and has been shown to play a role in regulating adipocyte differentiation and insulin signaling [20]. Overall, the different isoforms of p38 have distinct tissue distributions and functions but share a similar protein structure and function as stress-activated protein kinases that play critical roles in regulating cellular responses to stress and inflammation.

**Fig. 2:**
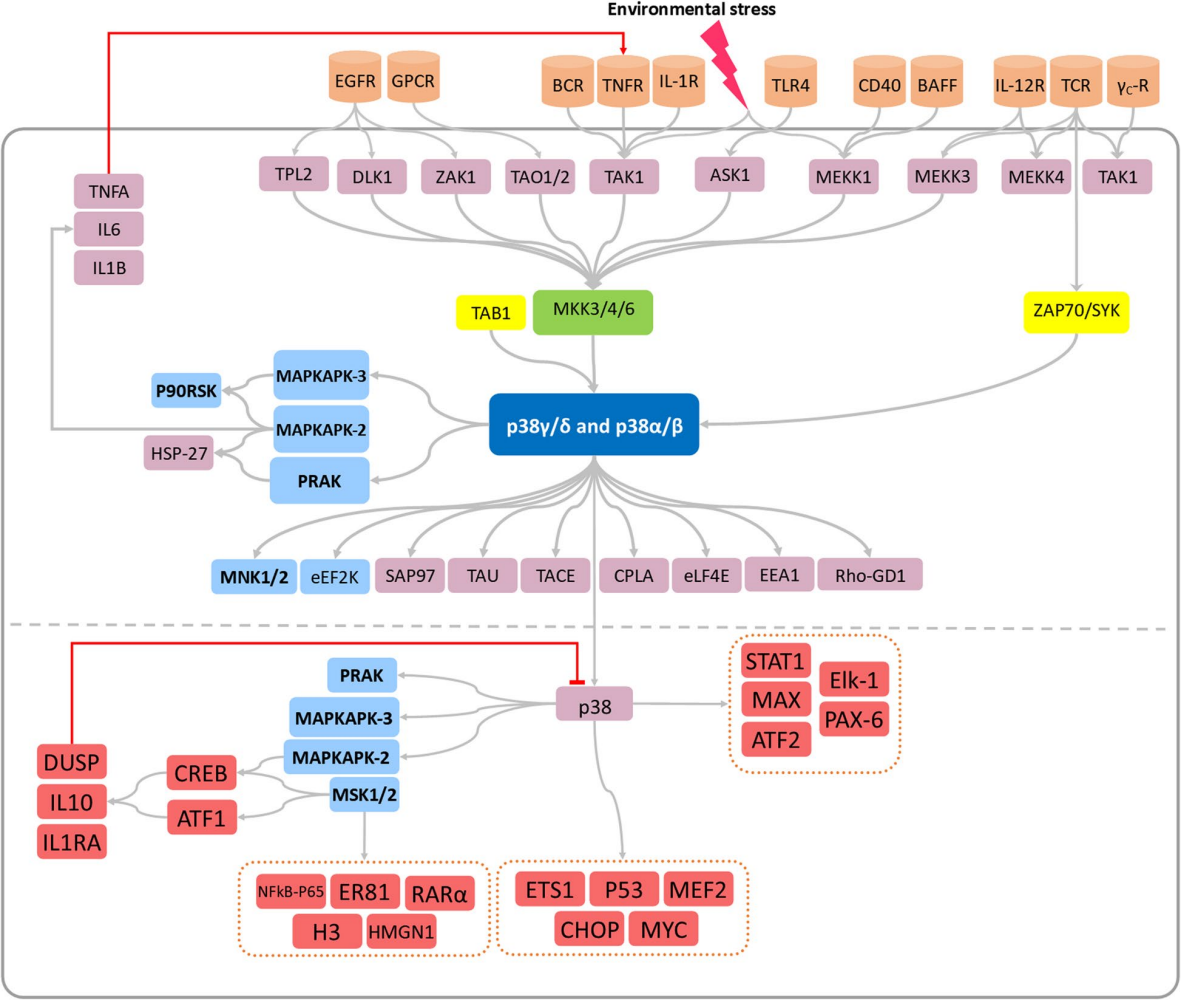
General p38 Signaling Pathways. The figure illustrates the general pathways of p38 signaling. Environmental signals are transmitted through cell receptors (colored orange) to multiple MAPK kinases (MAP3K, colored pink). These MAPKK kinases phosphorylate downstream MAPK kinases (MAPKK, colored green), which subsequently activate the p38 isoforms. In noncanonical pathways, MAPKs can be activated through phosphorylation by ZAP70/SYK or through interaction with TAB1. Once activated, p38 can either remain in the cytoplasm or translocate into the nucleus, leading to the activation of various transcription factors (colored red) and kinase proteins (colored blue)

The p38MAPK family includes highly conserved serine-threonine protein kinases with four isoforms, namely, α, β, γ, and δ. Although these proteins are activated downstream of various signals, they differ in terms of tissue distribution and activators/substrates [26]. A wide variety of stimuli, such as oxidative stress, inflammatory cytokines, and growth factors, stimulate several MAPK kinases (MEKK/MAP3K), which activate downstream kinases [26]. Alternatively, p38α and p38β can be activated in T cells in response to TCR (T-cell receptor) signaling, which is increased by T-cell costimulation, downstream of ζ-chain-associated protein kinase of 70 kilo Daltons (ZAP70) and spleen tyrosine kinase (SYK) protein (Fig. 2) [27].

Based on structural and sequence features as well as substrate specificities, the p38 MAPK family can be further divided into two subsets, p38α/p38β and p38γ/p38δ [10], with different effects on inflammation. For example, p38γ can inhibit c-Jun phosphorylation, but p38α promotes this process [28]. Additionally, p38γ may suppress the transcription factor activating protein-1 (AP-1), suggesting an anti-inflammatory effect of this isoform [29]. Furthermore, the expression of the β isoform appears restricted to T cells, while p38α is the most ubiquitously expressed isoform in different tissues [13, 30]. The expression levels of different p38 isoforms in COPD lung tissue have not been quantitatively studied. Therefore, p38α is considered the main studied isoform associated with COPD pathogenesis [15, 31].

Given the importance of the p38 signaling pathway and its observed overexpression in COPD, this section will focus on the role of p38 signaling in modulating different inflammatory cells that contribute to the pathogenesis of COPD.

### Dysregulation of p38 signaling

The dysregulation of the p38 signaling pathway has been linked to several diseases, including cancer, autoimmune disorders, and neurodegenerative diseases [32], highlighting the importance of comprehending the molecular mechanisms underlying p38 signaling.

In COPD, p38 signaling is upregulated in different cell types, such as bronchial epithelial cells, macrophages, and lymphocytes. This may contribute to chronic inflammation, tissue damage, and impaired repair in the lungs of COPD patients. Moreover, p38 signaling may also affect skeletal muscle structure and function in COPD, leading to sarcopenia and reduced exercise capacity. Oxidative stress has been shown to activate p38 signaling and cause muscle atrophy, fibrosis, and mitochondrial dysfunction in a mouse model of COPD [33].

## The role of p38 in COPD: from cell signaling to lung damage

The role of p38 in different inflammatory cells contributing to COPD pathogenesis is crucial and will be discussed in more detail in the following section. We aim to provide a comprehensive understanding of the specific role of p38 MAPK signaling in each cell type, including neutrophils, macrophages, T cells, and epithelial cells, which contribute to the development and progression of COPD.

### Epithelial cells

**Fig. 3:**
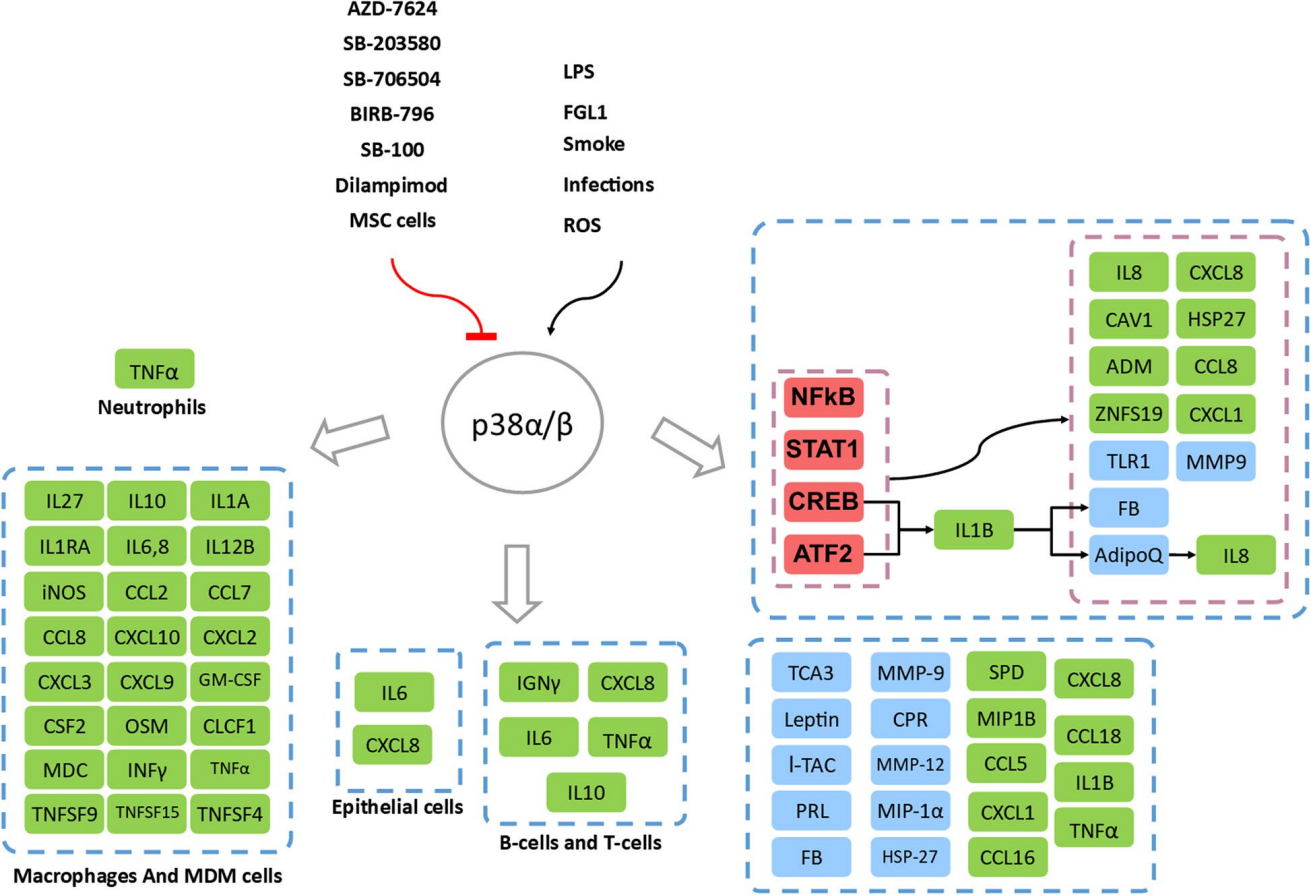
Association of p38 Signaling with COPD Pathogenesis. At the top of the figure, p38 activators and inhibitors are depicted. Downstream of p38 activation, cytokines are highlighted in green, while transcription factors are colored red. Other downstream biomolecules are represented in blue. The figure also highlights cell-specific downstream targets of p38 activation in various immune cell subtypes. However, for other molecules, the target cell types remain unclear

Cigarette smoke and other irritants stimulate lung epithelial cells to produce inflammatory mediators, such as TNF-α (tumor necrosis factor-alpha), IL-1β (interleukin 1 beta), IL-6, GM-CSF (granulocyte-macrophage colony-stimulating factor), and CXCL8 (IL-8) [34] (Table 1 and Fig. 3), resulting in TGF-β (transforming growth factor β) production by the small airway epithelium, which triggers local fibrosis and remodeling [35]. Additionally, it stimulates epithelial cells to release chemotactic factors that attract neutrophils, T-helper (Th) 1 (Th1/CD4+) cells, type 1 cytotoxic T cells (TC1/CD8+), and fibroblasts. Consequently, these inflammatory cells release proteases, growth factors, and proinflammatory cytokines, which promote chronic lung inflammation and structural changes [36].

**Table 1:** Genes and proteins that are affected downstream of p38 signaling within ex vivo models of COPD

**Gene**	**Source**	**Treatment**	**Case/Control**	**Up/Down**^**a**^	**Ref**
INFγ	CD8 cells (human)	S100 + IL12 + IL8	S100 + IL12 + IL8/ IL12 + IL8	Down-P	[23]
	MDM cells (human COPD)	Dexa^b^ + SB706504 + LPS	LPS/Dexa + SB706504 + LPS	Down-G	[37]
IL-6	Epithelial cells (human COPD)	S100 + polyinosinic: polycytidylic acid	S100 + I:P/I:P	Down-P	[23]
	Alveolar macrophages (AMs)	LPS + Dexa + BIRB-796	LPS + Dexa + BIRB-796/LPS	Down-P	[38]
	Macrophages (CS exposed rat)	MSC + CS	MSC + CS/CS	Down-G	[39]
	Guinea Pig AMs	SB 239063 + LPS	SB 239063 + LPS/LPS	Down-P	[40]
CXCL-8	AMs (human COPD)	BIRB-796 + LPS	BIRB-796 + LPS/LPS	Down-P	[41]
	Epithelial cells (human COPD)	S100 + I:P	S100 + I:P/I:P	Down-P	[23]
	PBMCs (COPD human)	LPS + Dexa + GW856553		Down-P	[42]
CCL-5	Epithelial cells (human COPD)	S100 + I:P	S100 + I:P/I:P	Down-P	[23]
	PBMCs (human COPD IV)	SB203580 + LPS	SB203580 + LPS/LPS	Down-P	[43]
TNFα	Blood neutrophils (human COPD)	S100 + LPS	S100 + LPS/basal	Down-P	[23]
	AM cells (human COPD)	BIRB-796 + LPS	BIRB-796 + LPS/LPS	Down-P	[41]
	Alveolar macrophages	LPS + Dexa + BIRB-796	LPS + Dexa + BIRB-796/LPS	Down-P	[38]
	Whole blood (COPD GOLD II)	SB-681323 + LPS	SB-681323 + LPS/Placebo + LPS	Down-P	[44]
	PBMCs (human COPD IV)	SB203580 + LPS	SB203580 + LPS/LPS	Up-P	[43]
IL-10	Macrophages (CS exposed rat)	MSC + CS	MSC + CS/CS	Up-G	[39]
	PBMCs (human COPD IV)	SB203580 + LPS	SB203580 + LPS/LPS	Up-P	[43]
	MDM cells (human COPD)	Dexa + SB706504 + LPS	LPS/Dexa + SB706504 + LPS	Down-G	[37]
	Alveolar macrophages	LPS + Dexa + BIRB-796	LPS + Dexa + BIRB-796/LPS	Down-P	[38]
iNOS	Macrophages (CS exposed rat)	MSC + CS	MSC + CS/CS	Down-G	[39]
COX-2	Macrophages (CS exposed rat)	MSC + CS	MSC + CS/CS	Down-G	[39]
PGE-2	Macrophages (CS exposed rat)	MSC + CS	MSC + CS/CS	Down-G	[39]
CCL7	MDM cells (human COPD)	Dexa + SB706504 + LPS	LPS/Dexa + SB706504 + LPS	Down-G	[37]
TNFSF-9	MDM cells (human COPD)	Dexa + SB706504 + LPS	LPS/Dexa + SB706504 + LPS	Down-G	[37]
CSF-2	MDM cells (human COPD)	Dexa + SB706504 + LPS	LPS/Dexa + SB706504 + LPS	Down-G	[37]
CCL-2	MDM cells (human COPD)	Dexa + SB706504 + LPS	LPS/Dexa + SB706504 + LPS	Down-G	[37]
TNFSF-15	MDM cells (human COPD)	Dexa + SB706504 + LPS	LPS/Dexa + SB706504 + LPS	Down-G	[37]
TNFSF-4	MDM cells (human COPD)	Dexa + SB706504 + LPS	LPS/Dexa + SB706504 + LPS	Down-G	[37]
IL-12B	MDM cells (human COPD)	Dexa + SB706504 + LPS	LPS/Dexa + SB706504 + LPS	Down-G	[37]
TNF	MDM cells (human COPD)	Dexa + SB706504 + LPS	LPS/Dexa + SB706504 + LPS	Down-G	[37]
CXCL-10	MDM cells (human COPD)	Dexa + SB706504 + LPS	LPS/Dexa + SB706504 + LPS	Down-G	[37]
IL-27	MDM cells (human COPD)	Dexa + SB706504 + LPS	LPS/Dexa + SB706504 + LPS	Down-G	[37]
CLCF-1	MDM cells (human COPD)	Dexa + SB706504 + LPS	LPS/Dexa + SB706504 + LPS	Down-G	[37]
OSM	MDM cells (human COPD)	Dexa + SB706504 + LPS	LPS/Dexa + SB706504 + LPS	Down-G	[37]
CCL-8	MDM cells (human COPD)	Dexa + SB706504 + LPS	LPS/Dexa + SB706504 + LPS	Down-G	[37]
IL-6	MDM cells (human COPD)	Dexa + SB706504 + LPS	LPS/Dexa + SB706504 + LPS	Down-G	[37]
IL-1A	MDM cells (human COPD)	Dexa + SB706504 + LPS	LPS/Dexa + SB706504 + LPS	Down-G	[37]
CXCL-9	MDM cells (human COPD)	Dexa + SB706504 + LPS	LPS/Dexa + SB706504 + LPS	Down-G	[37]
CXCL-2	MDM cells (human COPD)	Dexa + SB706504 + LPS	LPS/Dexa + SB706504 + LPS	Down-G	[37]
CXCL-3	MDM cells (human COPD)	Dexa + SB706504 + LPS	LPS/Dexa + SB706504 + LPS	Down-G	[37]
CXCL-1	MDM cells (human COPD)	Dexa + SB706504 + LPS	LPS/Dexa + SB706504 + LPS	Down-G	[37]
IL-8	MDM cells (human COPD)	Dexa + SB706504 + LPS	LPS/Dexa + SB706504 + LPS	Down-G	[37]
	Alveolar macrophages	LPS + Dexa + BIRB-796	LPS + Dexa + BIRB-796/LPS	Down-P	[38]
GM-CSF	Alveolar macrophages	LPS + Dexa + BIRB-796	LPS + Dexa + BIRB-796/LPS	Down-P	[38]
IL-1ra	Alveolar macrophages	LPS + Dexa + BIRB-796	LPS + Dexa + BIRB-796/LPS	Down-P	[38]
MDC	Alveolar macrophages	LPS + Dexa + BIRB-796	LPS + Dexa + BIRB-796/LPS	Down-P	[38]

^a^*Down* Downregulation, *Up* Upregulation, *P* Protein, *G* Gene

^b^*Dexa* Dexamethasone

In COPD patients, phospho-p38 is increased in small airway epithelia (compared to nonsmokers) [23] and in alveolar septa (compared to smokers and nonsmokers) [15]. Activation of p38 in epithelial cells of COPD patients induces local/systemic (pro) inflammatory signaling pathways and increases mucin and vascular endothelial growth factor (VEGF). p38-mediated mucin hypersecretion is accompanied by a decrease in the function of the tyrosine phosphatase SHP-1 in COPD patients [45]. p38 activation in bronchial epithelial cells can contribute to CXCR3-induced chemotaxis [46]. The expression of IL-8 and p38 was dramatically increased following exposure to oxidative and inflammatory agents such as H_2_O_2_, cytomix, and LPS (lipopolysaccharides) in the human bronchial epithelial cell line 16HBEBEAS-2B [47–50]. Meanwhile, p38 inhibition has been shown to reduce the production of pro-inflammatory cytokines such as IL-6, CXCL8, and CCL5 (Chemokine (C–C motif) ligand 5) from isolated lung epithelial cells of COPD patients [23] (Table 1 and Fig. 3). Reactive oxygen species can disrupt the integrity of the endothelial cell barrier through the p38-HSP27 (Heat Shock Protein 27)-dependent pathway, demonstrating the critical role of p38 signaling in the remodeling process of the lung epithelium [51]. C-reactive protein (CRP) and fibrinogen are systemic inflammation biomarkers that are associated with poor prognosis, increased mortality rate, and exacerbation periods in COPD patients [52]. CRP has been shown to be synthesized by respiratory epithelial cells [53]. The p38 inhibitors PH-797804 and losmapimod are suggested to regulate CRP and fibrinogen by inhibiting inflammatory pathways such as IL-1β downstream signaling [54–57].

Additionally, p38 is involved in the expression of TNF-α-induced VEGF in human airway smooth muscle cells (HASMC) [57], and balanced VEGF expression is critical to maintaining alveolar cell integrity [58]. Suppression of VEGF receptors leads to apoptosis and remodeling in alveolar cells and promotes emphysema-like pathology. Excessive VEGF expression may contribute to a COPD-like phenotype without emphysema [35, 59] (Table 1 and Fig. 3).

p38 signaling can also give rise to the development of secondary complications associated with COPD exacerbations, such as rhinovirus-induced VEFG production and influenza virus-induced interferon-stimulated gene expression of p38 in airway epithelial cells [60, 61]. The limited efficacy of corticosteroids in alleviating the chronic inflammation of human bronchial epithelial cells in asthma and COPD after an oxidative stress challenge has raised more attention to developing effective p38 inhibitors [62].

Another crucial finding is that in model mice, the premature senescence of airway epithelial cells (Clara cells) was induced by repeated exposure to a toxicant. This led to impaired epithelial regeneration and was accompanied by airway inflammation that was dependent on the activation of p38 MAPK. Moreover, in COPD patients, Clara cell senescence was observed to be accelerated and exacerbated by p38 MAPK, indicating its potential role in the development of COPD. This finding highlights the significance of Clara cell senescence and its interaction with p38 MAPK in the pathogenesis of COPD [63]. Several studies have reported similar observations regarding the role of premature senescence in COPD [64–66]. The collective body of research in this area underscores the importance of understanding the mechanisms underlying airway epithelial cell senescence and its implications in COPD.

Overall, these studies suggest that the p38 MAPK signaling pathway plays a crucial role in the progression of COPD in epithelial cells. Activation of the p38 MAPK pathway is associated with oxidative stress, cellular senescence, and inflammation, leading to the secretion of inflammatory proteins and the accumulation of senescent cells in the lungs. The dysregulation of TLRs [66], NOX4, Hsp70 [67, 68], miR-494-3p [64], CD147 [69], and ANGPTL4 [70] in COPD also involves the activation of the p38 MAPK signaling pathway.

A thorough investigation is necessary to fully understand the intricate molecular mechanisms that underlie the crucial role of the p38 MAPK signaling pathway in the pathogenesis of COPD and its related complications. It is imperative to find specific inhibitors and assess their safety and efficacy in clinical trials to develop effective treatments for COPD. Future research should be geared toward elucidating the underlying molecular mechanisms of the p38 MAPK signaling pathway in COPD pathogenesis and designing effective p38 inhibitors to alleviate chronic inflammation in bronchial and alveolar epithelial cells.

### Macrophages

Macrophages are important in COPD because they are involved in inflammatory and phagocytic processes, cytokine production and cellular interactions. Macrophages in COPD have been shown to have different phenotypes, functions and localizations, depending on the disease stage and severity [71]. Macrophages can contribute to the pathogenesis of COPD by causing oxidative stress, protease/antiprotease imbalance, apoptosis, antigen presentation and tissue destruction. Macrophages can also modulate the response to infection and inflammation by phagocytosing bacteria and apoptotic neutrophils [72]. The functions of macrophages can be impaired in COPD, leading to recurrent infections and exacerbations.

There are different kinds of macrophages in the respiratory system that have different physiological characteristics and unique functions. Alveolar macrophages (AMs) and IMs in the lung. AMs can be further divided into tissue-resident alveolar macrophages (TR-AMs) and monocyte-derived alveolar macrophages (Mo-AMs), depending on their origin and function. Furthermore, AMs can be subdivided into M1-like (proinflammatory) and M2-like (anti-inflammatory) phenotypes, depending on the stimuli they encounter [73]. M1-like macrophages contribute to the initiation and amplification of inflammation, while M2-like macrophages promote the resolution of inflammation and tissue repair.

Different stimuli, such as cigarette smoke, hypoxia, oxidative stress, and microbial infections, can modulate the phenotypes and functions of macrophages in COPD toward activation and polarization in inflammation and the immune response. p38 signaling is a pathway that regulates the expression of proinflammatory cytokines and mediators in macrophages, is activated by cigarette smoke and has increased activity in COPD alveolar macrophages. p38 signaling may contribute to inflammation, tissue damage and corticosteroid resistance in COPD.

Lung macrophage populations are elevated in both heavy smokers and COPD patients. Elevated levels of chemokines such as CCL2 and CXCL1, which are found to increase in the sputum and bronchoalveolar fluid of COPD patients, induce the recruitment of circulating monocytes [74] (Table 1 and Fig. 3). Furthermore, the percentage of alveolar macrophages expressing phospho-p38 is consistently higher in COPD patients [15, 23, 38]. A cell-type-specific analysis of the presence of phospho-p38 MAPK in the lung tissue of COPD patients, smokers, and nonsmokers revealed elevated levels in bronchial epithelial cells, macrophages, and CD20+ and CD8+ lymphocytes in COPD lungs but not in sputum and lung tissue neutrophils. Notably, p38 MAPK inhibition reduced proinflammatory mediator release in COPD lung CD8 cells and airway epithelia [23].

**Fig. 4:**
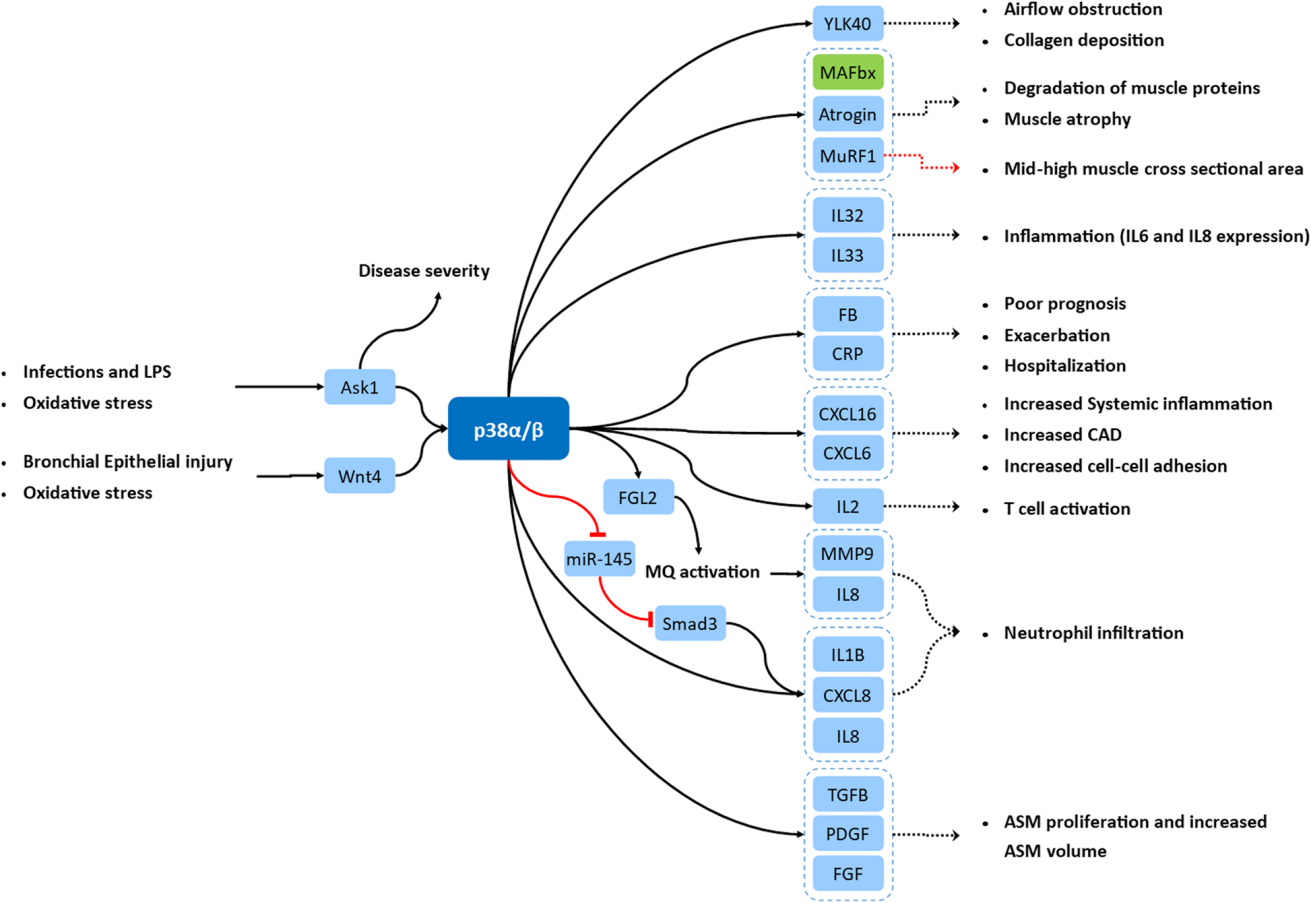
Clinical manifestations of the p38α/β signaling pathway. The activation of specific signaling pathways triggers the activation of the p38α/β signaling pathway. This pathway influences various cellular messengers, leading to the development of different phenotypes in diverse cell types

Activation of p38 can be initiated by the upregulation of cell receptors found upstream of the p38 signaling pathway. Fibrinogen-like protein 2 (FGL2) expression is upregulated in lung macrophages of COPD patients as well as in THP-1 cells, a human monocyte-macrophage line (Fig. 4). FGL2 is a transmembrane-anchored protein expressed on macrophages and endothelial cells that positively regulates proinflammatory cytokines such as IFN-γ (interferon-gamma) and TNF-α [75]. Conversely, FGL2 is also involved in LPS-induced macrophage activation through the p38 pathway. This interaction may stimulate the release of proinflammatory cytokines and enhance macrophage migration in the lung [76] (Fig. 3).

Despite numerous reports suggesting enhanced p38 phosphorylation in animal models after cigarette smoke exposure [77], p38 phosphorylation has been proposed to be more closely associated with COPD than with smoking [15] (Table 1 and Fig. 3). Research has proven that p38 inhibition can reduce the release of various proinflammatory cytokines, including TNF‐α and granulocyte-macrophage colony-stimulating factor (GM‐CSF), from COPD macrophages. Nonetheless, alveolar macrophages have been shown to be resistant to glucocorticoid anti-inflammatory therapies, and this phenomenon has been observed in both COPD patients and controls [23, 38, 78].

An in vitro study cultured the murine alveolar macrophage line (MH-S) with serum from COPD patients (GOLD stages of III/IV), which resulted in increased expression of CCL5 and decreased expression of IL-10 compared to healthy controls. However, secondary treatment of cells with the p38 inhibitor SB203580 had limited success in restoring impaired lymphocyte function and COPD-induced inflammation suppression, indicating the importance of p38-independent inflammatory mechanisms in the pathogenesis of COPD [43] (Table 2). Combining p38 inhibitors BIRB-796/SB706504 with corticosteroids in COPD patients can synergistically enhance the secretion of anti-LPS cytokines of alveolar macrophages, including IL-8, IL-6, TNF, IL-10, RANTES (Regulated on Activation, Normal T-cell Expressed and Secreted; CCL5), GM-CSF, IL-1β, IL-1ra, and MDC [37, 38, 78] (Table 1 and Fig. 3). This synergistic effect can be partially attributed to glucocorticoid-induced upregulation of MAPK phosphatase expression [79]. However, it should be noted that IL-1, IL-18, and CCL5 are resistant to coadministration of dexamethasone and SB706504 and thus do not show a substantial increase in expression [37]. Therefore, the therapeutic application of p38 inhibition is highly compromised due to the significant risk of inhibiting essential immune functions and its inefficacy on alveolar macrophages. Nonetheless, inhibiting the p38 pathway using the structurally distinct inhibitors VX745 and SCIO469 does not alter innate immune responses such as phagocytosis or efferocytosis by macrophages [80]. A study showed that although SB706504 could significantly reduce TNF-α expression in LPS-stimulated alveolar macrophages in COPD patients and smokers, the expression was further maximized by coadministration of SB706504 and dexamethasone [37]. In general, p38 activation is insensitive to corticosteroids, and combining a p38 inhibitor with a corticosteroid can synergistically enhance the anti-inflammatory effects [38].

**Table 2:** Genes and proteins that are affected downstream of P38 signaling within in vitro models of COPD

**Gene**	**Source**	**Treatment**	**Case/control**	**Up/down**^**a**^	**Ref**
IL-8	16HBE Cell line	H_2_O_2_/TNF-α + IL-1β + INFγ/LPS	H2O2/TNF-α + IL-1β + INFγ/LPS vs. baseline	Down-G	[50]
NFkB p65	16HBE Cell line	H2O_2_/TNF-α + IL-1β + INFγ/LPS	H2O2/TNF-α + IL-1β + INFγ/ baseline with LPS	Down-G	[50]
IL-10	MH-S cell line	GOLD III/IV serum + LPS + SB203580	GOLD III/IV serum + LPS + SB203580/ GOLD III/IV serum + LPS	Up-P	[43]
CCL-5	MH-S cell line	GOLD IV COPD serum + LPS + SB203580	GOLD III and IV COPD serum + LPS + SB203580/ GOLD III and IV COPD serum + LPS	Down-P	[43]
TNFα	MH-S cell line	GOLD III and IV COPD serum + LPS + SB203580	GOLD III and IV COPD serum + LPS + SB203580/ GOLD III and IV COPD serum + LPS	Down-P	[43]
GRP-78	A549 cells	SB203580 + CS	SB203580 + CS/CS	Down-G&P	[81]

^a^*Down* Downregulation, *Up* Upregulation, *P* Protein, *G* Gene

Smokers with a high MMP-9/TIMP-1 ratio have been found to have a positive correlation with the annual decline in forced expiratory volume in 1 s (FEV1%), forced vital capacity (FVC%), and MMEF%pred. Moreover, inhibition of p38 MAPK and ERK suppressed the production of MMP-9 in alveolar macrophages (AMs) from smokers. Furthermore, Guan et al. [82] reported that MMP-12 contributes to the proliferation of mouse macrophages by regulating the ERK/P38 MAPK signaling pathway. These findings suggest that the MMP-9/TIMP-1 ratio could be a useful predictor of future risk of COPD among smokers with AHR. Additionally, previous studies have investigated the role of MMP-9/TIMP-1 imbalance in various lung diseases, including COPD and asthma [83].

Alveolar macrophages (AMs) and monocyte-derived macrophages (MDMs) from COPD patients showed lower levels of bacterial phagocytosis and efferocytosis than those from healthy controls. It was found that p38 MAPK inhibition impaired bacterial phagocytosis by M1 macrophages from COPD patients but enhanced efferocytosis by M2 macrophages from all groups [80], while Vij et al. [84] found that cigarette smoke activated p38 MAP kinase and inhibited autophagy in macrophages, leading to increased inflammation and impaired phagocytosis. However, none of the inhibitors, including p38 MAPK inhibitors, altered bacterial internalization or early intracellular bacterial killing in AM or MDM [80]. Additionally, in an in vitro model of human alveolar macrophage (AM) smoke exposure, cigarette smoke activated p38 MAP kinase in GM-CSF-derived macrophages (GM-MOs) and enhanced IL8 production while inhibiting phagocytosis, similar to the phenotype of smokers’ Ams [85].

### Neutrophils

Neutrophils are involved in the lung response to various infections and diseases, such as tuberculosis, acute lung injury, and chronic pulmonary diseases. Depending on the context, neutrophils can have beneficial or detrimental effects on lung tissue and function. The role of neutrophils in the lung is complex and dynamic and depends on the type, duration, and severity of the infection or injury. Neutrophils can either protect or damage lung tissue by regulating the balance between inflammation and resolution, defense and repair, or clearance and fibrosis. Based on these functions, neutrophils in the lung can be classified into different kinds according to their phenotype, activation state, origin, or location. Lung-resident neutrophils, blood-derived neutrophils, senescent (aged) neutrophils, and N1 and N2 neutrophils are subsets of neutrophils that can be found in the respiratory system [86, 87].

Lung-resident neutrophils are a subset of neutrophils that reside in the lung under steady-state conditions. They have a unique immunosuppressive phenotype that protects the lungs against damage from overexaggerated responses to injury or infection. They express high levels of CXCR4 and CD11b but low levels of CD62L. They are maintained by high levels of interleukin-6 (IL-6) and prostaglandin E2 (PGE2) in the lung [88, 89].

Blood-derived neutrophils are neutrophils that migrate from the blood into the lung upon inflammatory stimuli. They have a proinflammatory phenotype that helps fight infection and clear debris. They express high levels of CD62L and CD16 but low levels of CXCR4. They are recruited by chemokines such as CXCL1, CXCL2, CXCL5, CXCL8, CCL2, CCL3, CCL4, CCL5, CCL7, and CCL8 [90].

Senescent neutrophils are also a subset of neutrophils that have a prolonged lifespan due to inflammatory signals or oxidative stress. Senescence refers to a state of irreversible growth arrest and altered cellular function. They have an altered phenotype that makes them more prone to apoptosis or NET formation. They express high levels of CD44 and CD64 but low levels of CD16 and CD62L. They are cleared by macrophages or efferocytosis [91, 92].

N1 and N2 neutrophils are two subsets of neutrophils that have different polarization states depending on the cytokine environment. N1 neutrophils are pro-inflammatory and produce high levels of TNF-α, interferon gamma (IFN-γ), IL-12, IL-23, IL-1β, IL-6, and IL-8. N2 neutrophils are anti-inflammatory and produce high levels of IL-10, TGF-β, arginase 1 (ARG1), VEGF, and IL-4. N1 neutrophils have antitumorigenic effects by killing tumor cells, stimulating cytotoxic T cells, and inhibiting angiogenesis. N2 neutrophils have protumorigenic effects by promoting tumor growth, metastasis, and immunosuppression. N1 neutrophils are involved in the pathogenesis of autoimmune diseases such as rheumatoid arthritis, systemic lupus erythematosus, and multiple sclerosis by producing inflammatory mediators, forming NETs, and activating autoreactive T cells. N2 neutrophils are involved in the resolution of inflammation and tissue repair by producing anti-inflammatory mediators, clearing apoptotic cells, and inducing regulatory T cells. N1 neutrophils are essential for the clearance of intracellular pathogens such as Mycobacterium tuberculosis, Leishmania donovani, and Salmonella typhimurium by enhancing phagocytosis, ROS production, and cytokine secretion. N2 neutrophils are detrimental to the host defense against these pathogens by impairing phagocytosis, ROS production, and cytokine secretion [93–95].

In COPD, neutrophils represent the predominant subtype of white blood cells involved in the inflammatory response. However, it is important to recognize that neutrophils can exhibit functional heterogeneity, indicating the presence of different subtypes or phenotypes based on their activation status and functions within the disease context [96]. One subtype identified in COPD is the “primed” or “activated” neutrophils, characterized by an enhanced ability to release inflammatory mediators and reactive oxygen species. These neutrophils display an altered phenotype with increased expression of specific surface markers, such as CD11b and CD66b [97]. Another subtype is the “senescent” neutrophils found in the airways of COPD patients, which are characterized by impaired apoptosis and reduced clearance by macrophages, leading to their accumulation in the lungs [98].

Different subtypes of neutrophils contribute to the inflammatory processes and tissue damage observed in COPD. However, it is important to note that neutrophil counts alone cannot solely determine the severity or progression of COPD. A comprehensive evaluation of the disease requires clinical assessment, considering symptoms, exacerbation history, and imaging findings. Additionally, it is important to acknowledge that neutrophil counts can vary and fluctuate among individuals with COPD, influenced by factors such as disease stage, exacerbation status, and individual patient characteristics [1].

Neutrophil counts in COPD display heterogeneity across different respiratory system locations. Inflammation, immune response, and bacterial infection contribute to increased neutrophil counts in the airways, bronchi, bronchial epithelium [99], mucous glands, BAL fluid [98, 100], and sputum samples of COPD patients [98, 101–103]. Neutrophil counts in peripheral blood can be either increased or normal, depending on the disease phase [102]. The systemic nature of neutrophilic inflammation in COPD suggests involvement beyond localized regions. In the lung parenchyma and alveoli, neutrophil infiltration occurs in regions of tissue damage and inflammation, with variations in counts linked to disease severity and exacerbations [98].

Multiple studies have provided evidence of increased p38 signaling in alveolar macrophages, epithelial cells, and lymphocytes within the lungs of individuals with COPD, while neutrophils do not exhibit the same upregulation [15, 23]. This suggests that p38 signaling may have distinct roles and effects across different cell types. However, other research has demonstrated the involvement of p38 signaling in neutrophil activation, migration, and survival in COPD. Notably, p38 signaling is responsible for mediating the release of neutrophil elastase, a protease implicated in pulmonary damage and inflammation [16]. Furthermore, p38 signaling has been identified as a regulator of CXCR2 expression, a chemokine receptor that guides neutrophil recruitment to the airways in COPD [104].

COPD lung neutrophils are a significant source of CXCL8 (IL8) secretion, which is the primary cytokine involved in the recruitment neutrophils during COPD pathogenesis [105]. After localization, neutrophils contribute to alveolar destruction and airway remodeling by releasing various metalloproteases, such as MMP-8 and MMP-9, as well as several other proteinases, including elastase, cathepsin-G, and proteinase-3. Although p38 isoforms have been reported in several immune cells [25], the dual phosphorylation and activation of p38 have not been observed in resident sputum neutrophils in COPD patients. It seems that the activation of this kinase is not necessary for the proinflammatory activity of lung neutrophils in COPD [23]. The selective inhibitor of p38α and p38β, SB100, was not effective in suppressing the proinflammatory cytokines released from lung neutrophils [23]. Additionally, lung neutrophils exhibit low expression of the glucocorticoid receptor [106] (Table 1 and Fig. 4). This altered behavior of lung neutrophils suggests that specific anti-neutrophil therapies beyond broad anti-inflammatory agents are necessary to manage COPD complications.

Therefore, the role of p38 signaling in neutrophils in COPD is of paramount importance, contingent upon the specific context and activating stimuli. The inhibition of p38 signaling holds promise in attenuating inflammation and averting tissue damage in COPD. However, it is imperative to acknowledge the potential adverse effects, including compromised host defense mechanisms and hindered bacterial clearance, associated with p38 signaling inhibition. Consequently, further investigation is warranted to gain a comprehensive understanding of the precise mechanisms and consequences of p38 signaling in neutrophils within the context of COPD.

### Lymphocytes

Recent studies have explored the relationship between inflammatory cell infiltration and COPD, with particular attention given to CD8+ (TC1) cells [107]. These studies have found that the increased numbers of these cells in COPD patients are the only significant difference in inflammatory cell infiltration between asymptomatic smokers and those with COPD [108, 109]. Furthermore, this infiltration is correlated with alveolar destruction levels and the intensity of airflow obstruction [107]. In addition to CD8+ (TC1) cells, upregulation of phospho-p38 has also been associated with the severity of lung function decline and the number of infiltrating CD8+ T-lymphocytes in alveolar walls [15].

The therapeutic significance of p38 inhibitors has been emphasized, given the observation that selective inhibition of p38α and β can suppress IL-2 production in lymphocytes, making these isoforms highly relevant to CD8+ T-cell function [110]. In addition, SB100-induced p38 inhibition reduces proinflammatory cytokine production in lung and blood CD8+ cells in COPD patients, while p38 inhibition significantly reduces IFN-γ release from COPD lung-isolated CD8+ cells [23]. Nevertheless, phospho-p38 expression in lymphocytes found in the peripheral airway submucosa was comparable in COPD patients, smokers, and nonsmokers (Table 1 and Fig. 3). In COPD patients, the inhibitory effect of glucocorticoids on IFN-γ release from bronchi-alveolar lymphocytes is reduced compared to controls [111]. Moreover, IFN-γ induces alveolar macrophages to secrete cytokines in a glucocorticoid-insensitive manner through STAT1 (signal transducer and activator of transcription 1) activation in COPD patients [112]. Thus, the inhibition of p38 signaling in lung CD8+ cells could overcome glucocorticoid insensitivity mechanisms, such as IFN-γ production from CD8+ cells and subsequent STAT1-mediated cytokine production from macrophages. Interestingly, the combination of a low dose of a selective p38 inhibitor, losmapimod (GW856553), with dexamethasone has been shown to have a synergistic effect on the suppression of CXCL8 release from peripheral blood mononuclear cells (PBMCs) of COPD patients [42] (Table 1 and Fig. 3).

### Smooth muscle cells

Smooth muscle cells (SMCs) are a type of involuntary muscle cells that are found in various organs and tissues, such as the blood vessels, the digestive tract, the urinary bladder, and the eye. Smooth muscle cells can contract and relax to regulate the flow of fluids and substances in the body.

In the lung, SMCs are mainly found in the walls of the airways, such as the trachea, bronchi, and bronchioles. They help control the diameter of the airways and the resistance to airflow. Smooth muscle cells in the lung can also respond to various stimuli, such as hormones, neurotransmitters, inflammatory mediators, and environmental factors. For example, smooth muscle cells can contract in response to histamine, a chemical released during allergic reactions, causing bronchoconstriction and asthma symptoms.

Smooth muscle cells in the lung are also involved in some diseases and disorders, such as COPD, pulmonary hypertension, and lung cancer. In COPD, smooth muscle cells in the airways become hypertrophied (enlarged) and hyperplastic (increased in number), contributing to airway remodeling and obstruction. In pulmonary hypertension, smooth muscle cells in the pulmonary arteries become proliferative and migratory, leading to vascular remodeling and increased blood pressure in the lungs [113].

The role of p38 signaling in SMCs in COPD is not fully understood, but some studies have suggested that it may be involved in the pathogenesis and progression of the disease. In SMCs, p38 MAPKs may have different roles depending on the location and stage of the disease. In airway SMCs, p38 MAPKs may contribute to airway inflammation and remodeling by inducing the production of proinflammatory cytokines, such as IL-8. In addition, p38 MAPKs may regulate the contractility and proliferation of airway SMCs, which may affect airway hyperresponsiveness and obstruction. In vascular SMCs, p38 MAPKs may be involved in pulmonary hypertension, a common complication of COPD. p38 MAPKs may promote the proliferation and migration of vascular SMCs, leading to vascular remodeling and increased pulmonary vascular resistance [114]. Therefore, p38 signaling in SMCs in COPD may have both beneficial and detrimental effects on the disease outcome. Inhibiting p38 MAPKs may have anti-inflammatory and bronchodilatory effects, but it may also impair host defense and bacterial clearance.

SMCs are involved in the inflammation associated with COPD [115]. SMCs release macrophage activating factors and chemokines, such as IL-8 and GM-CSF [116, 117]. Furthermore, the release of inflammatory mediators from HASMCs can cause insensitivity to corticosteroids. In contrast to corticosteroids, resveratrol can effectively reduce IL-8 and GM-CSF release or moderately reduce VEGF release from HASMCs in COPD patients. Therefore, resveratrol may be a preferred therapy for COPD over corticosteroids [118]. A study demonstrated that resveratrol’s p38MAPK blockage can be implemented in corticosteroid-insensitive SMCs in COPD [119].

Additionally, in COPD, SMCs in the airways become hypertrophied (enlarged) and hyperplastic (increased in number), contributing to airway remodeling and obstruction [120]. In addition, SMCs in COPD show altered metabolism and mitochondrial function, which may affect their growth, survival, and inflammation [121].

### Endothelial cells

Endothelial cells play a crucial role in the lung by lining the inner surface of blood vessels and lymphatic vessels. They are involved in various important functions, including gas exchange, vascular permeability, angiogenesis, inflammation, and fibrosis. These cells form a thin barrier between the blood and the alveoli, enabling the exchange of oxygen and carbon dioxide. Additionally, endothelial cells produce nitric oxide, a vasodilator that regulates blood flow and pressure in the lung. One of the key functions of endothelial cells is controlling the movement of fluid and molecules across the blood vessel wall. They can adjust their permeability by altering their shape, junctions, and cytoskeleton. Moreover, endothelial cells secrete factors that influence the permeability of other cells, such as epithelial cells and fibroblasts [122]. Endothelial cells also play a vital role in angiogenesis, which is the formation of new blood vessels from preexisting ones. This process is essential for normal lung development, growth, and repair. In response to various environmental signals, such as hypoxia, growth factors, and cytokines, endothelial cells can sense and respond by proliferating, migrating, and differentiating to form new vessels. Furthermore, endothelial cells actively participate in the inflammatory response within the lung. They accomplish this by expressing adhesion molecules, chemokines, and cytokines that recruit and activate leukocytes. Additionally, endothelial cells interact with immune cells such as macrophages and dendritic cells to modulate their function [123]. In the context of pulmonary fibrosis, endothelial cells contribute to the pathological process through a phenomenon called endothelial-to-mesenchymal transition (EndMT). This process involves endothelial cells losing their endothelial identity and acquiring mesenchymal characteristics. EndMT can lead to the loss of capillaries and the accumulation of myofibroblasts and extracellular matrix in the lung, ultimately contributing to fibrosis.

In individuals with COPD, there is evidence of shortened telomerase activity and reduced telomere length in pulmonary endothelial cells. These findings suggest the presence and increased accumulation of senescent endothelial cells in COPD patients [124]. The senescence observed in the pulmonary endothelium of COPD patients can be attributed to chronic exposure to oxidative stress and the persistence of inflammation. These pathological conditions worsen the release of inflammatory cytokines from endothelial cells, contributing to the senescence of endothelial function in the lungs of individuals with COPD [124, 125]. Furthermore, COPD patients demonstrate reduced levels of endothelial PAS domain-containing protein 1 (EPAS1) messenger RNA (mRNA) and an increase in EPAS1 promoter methylation, which correlates with elevated TLR4 expression. Through in vitro experimentation, it was observed that TLR4 inhibits EPAS1 mRNA expression while concurrently promoting promoter methylation in endothelial cells. These findings substantiate the notion that TLR4 overexpression within endothelial cells contributes to the progression of COPD by downregulating EPAS1 expression [126].

Exposure to cigarette smoke, a prominent risk factor for COPD, disrupts multiple pathways in endothelial cells, leading to endothelial cell dysfunction and inflammation. Notably, the p38 MAPK pathway plays a significant role in mediating endothelial cell dysfunction and inflammation in the context of COPD. The involvement of p38 MAPK in driving endothelial cell dysfunction and inflammation underscores its critical role in the pathogenesis of COPD. Further investigations are warranted to unravel the underlying mechanisms by which p38 MAPK contributes to these processes in endothelial cells. This knowledge will pave the way for the development of targeted therapies that can modulate this pathway and potentially alleviate endothelial dysfunction and inflammation in COPD.

### Fibroblasts

Fibroblasts represent a diverse population of cells that play a crucial role in maintaining the structural integrity of the lung under both normal and pathological conditions. Their primary function involves the synthesis and remodeling of the extracellular matrix (ECM), which provides support to the lung tissue. Additionally, they actively participate in processes such as wound healing, inflammation, and fibrosis. Various types of cells closely associated with fibroblasts include resident fibroblasts, myofibroblasts, pericytes, mesenchymal stem cells (MSCs), and fibrocytes. Among these, resident fibroblasts constitute the predominant cell type within the lung interstitium and can be categorized further based on their location, morphology, function, and specific cell surface markers, such as Thy-1 [127, 128]. Myofibroblasts, on the other hand, are activated fibroblasts that exhibit heightened contractility and secrete substantial amounts of ECM proteins such as collagen and fibronectin. While they play a critical role in wound closure and tissue repair, dysregulation of myofibroblasts can lead to fibrosis [129]. Pericytes, which are contractile cells surrounding capillaries and microvessels in the lung, regulate vascular tone, permeability, and angiogenesis. In response to certain stimuli, pericytes can differentiate into myofibroblasts or other types of mesenchymal cells [128]. Mesenchymal stem cells represent a multipotent cell population capable of self-renewal and differentiation into various lineages, including fibroblasts, myofibroblasts, adipocytes, chondrocytes, and osteoblasts. These cells possess immunomodulatory and anti-inflammatory properties, and they can migrate to sites of injury or inflammation. [130]. Last, fibrocytes are circulating cells derived from the bone marrow that express both hematopoietic and mesenchymal markers. In response to inflammatory signals, fibrocytes can migrate to the lung and differentiate into fibroblasts or myofibroblasts. Their role in lung fibrosis involves enhancing ECM deposition and promoting fibroblast proliferation [130].

In COPD, fibroblasts play a significant role in various pathological mechanisms that contribute to disease progression and severity. These mechanisms include EMT, senescence, protease-antiprotease imbalance, and cell‒cell interactions. Additionally, the p38 signaling pathway plays a crucial role in regulating the function and phenotype of fibroblasts in COPD.

EMT is particularly implicated in smoking-related COPD, as it can lead to the loss of epithelial barrier function, increased inflammation, and enhanced deposition of the ECM [131, 132]. The p38 signaling pathway is one of the key pathways involved in mediating EMT in response to cigarette smoke and other stimuli [133].

Senescent fibroblasts, characterized by reduced proliferation, increased proinflammatory and profibrotic mediators, and impaired ECM remodeling, also contribute to COPD pathology [134, 135]. The p38 signaling pathway is involved in inducing and maintaining senescence in fibroblasts through various mechanisms, including the DNA damage response, oxidative stress, and cytokine signaling [136, 137].

Protease-antiprotease imbalance refers to an alteration in the balance between proteases (enzymes that breakdown proteins) and antiproteases (proteins that inhibit proteases), leading to excessive degradation of the ECM and tissue damage. Fibroblasts can produce both proteases (such as matrix metalloproteinases, or MMPs) and antiproteases (such as plasminogen activator inhibitors, or PAIs) and can modulate their activity in response to various stimuli. In COPD, fibroblasts may exhibit an altered protease-antiprotease balance that favors ECM destruction. The p38 signaling pathway is involved in regulating the expression and activity of proteases and antiproteases in fibroblasts through transcriptional and posttranslational mechanisms [138, 139].

In severe COPD, the uncoupling of pericytes from the microvasculature, as well as their migration toward the larger pulmonary arteries, are significant pathogenic factors contributing to emphysema and pulmonary hypertension (PH) [140]. Furthermore, cigarette smoke extract (CSE) has been found to activate the proliferation of pulmonary fibroblasts through p38 MAPK signaling, resulting in airway remodeling in COPD [141].

Treatment of lung fibroblasts obtained from former and current smokers with CSE revealed that CSE exposure affects the cell cycle by reducing the S phase and increasing the G1 and G2/M phases, increasing the expression of p53 and p21, activating the p38 and ERK 1/2 pathways, and augmenting IL-8 release. Inhibitors of p38 and ERK 1/2 reversed the effects of CSE on the cell cycle and IL-8 release, suggesting that cigarette smoke exposure may hamper the reparative potential of lung fibroblasts by altering the expression of p53 and p21 and the progression of the cell cycle, with a potential role for p38 and ERK 1/2 signaling [142].

In summary, the findings from these studies suggest that p38 MAPK signaling plays a critical role in regulating lung fibroblast function and may be involved in the pathogenesis of lung diseases such as COPD. Inhibition of p38 signaling has the potential to be a therapeutic strategy in reducing fibroblast-mediated inflammation and airway remodeling in these diseases.

## Targeting p38 as a therapeutic strategy for COPD treatment

Several p38 inhibitors have been developed and tested in cellular and animal models of COPD, as well as in clinical trials with COPD patients. These inhibitors have shown potent anti-inflammatory effects in vitro and in vivo, often greater than those of corticosteroids or other kinase inhibitors. In the subsequent sections, we will provide an in-depth analysis of the findings derived from in vitro, ex vivo, and in vivo investigations, as well as insights obtained from clinical trials.

### Insights from in vitro, ex vivo, and in vivo animal models

There has been a growing interest in the clinical efficacy of p38 inhibition for COPD complications. P38 MAPK inhibitors may be a promising treatment choice for COPD. The majority of p38 inhibitors target the alpha and beta isoforms, which have been implicated in the inflammatory processes associated with COPD. The inhibitors include PH-797804, SB-681323 (dilmapimod), SB-239063, RV568, and GW856553 [15, 31, 143, 144]. Isoform-selective p38 MAPK inhibitors exert their effects by binding to the ATP-binding site and selectively inhibiting the alpha and beta isoforms [145].

In an LPS-challenged mouse model, RV568 affected both α and γ isoforms of p38, significantly reducing chemotactic mediators of Th1 and Th17 cytokines, airway remodeling markers, and malondialdehyde, an oxidative stress biomarker [146]. However, as RV568 has a considerable impact on the SRC kinase family (steroid receptor coactivator) and HCKs (hematopoietic cell kinase) in particular, these results cannot be solely attributed to p38 inhibition [146] (Table 3 and Fig. 3). Additionally, human mesenchymal stem cells were used to ameliorate airway inflammation and emphysema by downregulating cyclooxygenase-2 expression (COX-2) and prostaglandin E2 (PGE2) production through the inhibition of p38 and ERK [39] (Fig. 3 and Table 1). Studies have shown that inhibitors of P38 MAPK can effectively reduce airway inflammation, with the p8 MAPK inhibitor SB706504 demonstrating a decreased LPS-induced release of TNF-α from macrophages in vitro. A study demonstrated that simultaneous exposure of PBMCs from COPD patients to both dexamethasone and GW856553 resulted in an increased reduction in CXCL8 levels, suggesting restoration of COPD sensitivity to corticosteroids [42]. Therefore, despite the inadequate response of COPD patients to conventional anti-inflammatory treatments such as corticosteroids, coadministration of p38 inhibitors and corticosteroids may show more promising therapeutic effects.

**Table 3:** Genes and proteins that are affected downstream of p38 signaling within in vivo models of COPD

**Gene**	**Source**	**Treatment**	**Case/control**	**Up/down**^**a**^	**Ref**
MMP-12	COPD model in Mice (BALF sample)	CS + LPS	CS + LPS/PBS	Up-P	[147]
	Emphysema mice model (BALF sample)	CS + LPS	Emphysema mice/WT	Up-P	[147]
	Lung tissue (emphysema mice model)	CS + SB203580	CS + SB203580/CS	Down-G	[148]
IL-16	COPD model in Mice (BALF sample)	CS + LPS	CS + LPS/PBS	Up-P	[147]
	Emphysema mice model (BALF sample)	CS + LPS	Emphysema mice/WT	Up-P	[147]
IL-27	COPD model in Mice (BALF sample)	CS + LPS	CS + LPS/PBS	Up-P	[147]
	Emphysema mice model (BALF sample)	CS + LPS	Emphysema mice/WT	Up-P	[147]
Leptin	COPD model in Mice (BALF sample)	CS + LPS	CS + LPS/PBS	Up-P	[147]
	Emphysema mice model (BALF sample)	CS + LPS	Emphysema mice/WT	Up-P	[147]
TCA-3	COPD model in Mice (BALF sample)	CS + LPS	CS + LPS/PBS	Up-P	[147]
	Emphysema mice model (BALF sample)	CS + LPS	Emphysema mice/WT	Up-P	[147]
KC (CXCL-1)	COPD model in Mice (BALF sample)	CS + LPS	CS + LPS/PBS	Up-P	[147]
	Emphysema mice model (BALF sample)	CS + LPS	Emphysema mice/WT	Up-P	[147]
	Lung tissue (emphysema mice model)	CS + SB203580	CS + SB203580/CS	Down-G	[55]
CRP	Emphysema mice model (BALF sample)	CS + LPS	Emphysema mice/WT	Up-P	[147]
	Circulating chemokine (COPD GOLD II)	GW856553	GW856553/Plasebo	Down-P	[149]
	Human COPD (GOLD II/III)	PH-797804	PH-797804/Placebo	Down-P	[55]
ITAC	Emphysema mice model (BALF sample)	CS + LPS	Emphysema mice/WT	Up-P	[147]
Adiponectin	Emphysema mice model (BALF sample)	CS + LPS	Emphysema mice/WT	Up-P	[147]
Prolactin	Emphysema mice model (BALF sample)	CS + LPS	Emphysema mice/WT	Up-P	[147]
uPAR	Emphysema mice model (BALF sample)	CS + LPS	Emphysema mice/WT	Up-P	[147]
FAM-174A	Whole blood (Human COPD)	Dilmapimod	Dilmapimod/placebo	Down-G	[54]
ADM	Whole blood (Human COPD)	Dilmapimod	Dilmapimod/placebo	Down-G	[54]
CCL-8	Whole blood (Human COPD)	Dilmapimod	Dilmapimod/placebo	Down-G	[54]
CXCL-1	Whole blood (Human COPD)	Dilmapimod	Dilmapimod/placebo	Down-G	[54]
IL-1β	Whole blood (Human COPD)	Dilmapimod	Dilmapimod/placebo	Down-G	[54]
	Lung tissue (emphysema mice model)	CS + SB203580	CS + SB203580/CS	Down-G	[55]
TLR-1	Whole blood (Human COPD)	Dilmapimod	Dilmapimod/placebo	Down-G	[54]
ZNF-519	Whole blood (Human COPD)	Dilmapimod	Dilmapimod/placebo	Down-G	[54]
MMP-9	Whole blood (Human COPD)	Dilmapimod	Dilmapimod/placebo	Down-G	[54]
	Guinea Pig BAL fluid	SB 239063 + LPS	SB 239063 + LPS/LPS	Down-P	[40]
COX-2	CS exposed rat	MSC + CS	MSC + CS/CS	Down-P	[39]
PGE-2	CS exposed rat	MSC + CS	MSC + CS/CS	Down-P	[39]
CXCL-8	Septum (human COPD)	-	COPD/healthy	Up-G&P	[143]
MMP-2	Lung tissue (emphysema mice model)	CS + SB203580	CS + SB203580/CS	Down-G	[55]
TNFα	Lung tissue (emphysema mice model)	CS + SB203580	CS + SB203580/CS	Down-G	[55]
	alveolar macrophages (COPD GOLD IV)	LPS + AZD7624	COPD/healthy	Down-P	[144]
	Rat whole blood	SB-239063 + LPS	SB 239063 + LPS/LPS	Down-P	[40]
MIP-1α	Lung tissue (emphysema mice model)	CS + SB203580	CS + SB203580/CS	Down-G	[55]
IL-6	Lung tissue (emphysema mice model)	CS + SB203580	CS + SB203580/CS	Down-G	[55]
	AMs (COPD GOLD IV)	LPS + AZD7624	COPD/healthy	Down-P	[144]
	Circulating chemokine (COPD GOLD II)	GW856553	GW856553/Placebo	Down-P	[149]
	Guinea Pig BAL fluid	SB-239063 + LPS	SB 239063 + LPS/LPS	Down-P	[40]
Fibrinogen	Circulating chemokine (COPD GOLD II)	GW856553	GW856553/Placebo	Down-P	[149]
IL-8	Circulating chemokine (COPD GOLD II)	GW856553	GW856553/Placebo	Down-P	[149]
HSP-27	Circulating chemokine (COPD GOLD II)	SB-681323	SB-681323/Placebo	Down-P	[44]

^a^*Down* Downregulation, *Up* Upregulation, *P* Protein, *G* Gene

The coadministration of dexamethasone and the p38 MAPK inhibitor BIRB-796 resulted in greater cytokine suppression in alveolar macrophages than each drug alone. This pharmacological synergism may allow for the use of smaller doses of each drug, aiming to optimize efficacy and minimize adverse effects. Thus, this therapeutic approach may provide insight into the potential of p38 MAPK inhibition as a real-life treatment for COPD, an area that is currently surrounded by uncertainties [150].

In vivo experiments in mice have revealed that the p38 MAPK inhibitor SD-282 inhibits smoke-induced increases in the number of bronchoalveolar lavage (BAL) neutrophils and macrophages, while steroids have negligible effects. An investigation on the effect of CSE in mice showed that the expression of IL-33 in bronchial endothelial cells and peripheral blood mononuclear cells (PBMCs) of COPD mice is upregulated, leading to enhanced production of IL-6 and interleukin-8 (IL-8). Importantly, the study demonstrated that p38 MAPK inhibitors effectively reduced the secretion of IL-6 and IL-8 in PBMCs and controlled systemic inflammation in COPD [151].

In another study, the association between oxidative stress and structural alterations in muscles in elastase-induced emphysema mouse models was investigated, focusing on the effect of p38 inhibitors. Activation of the p38 MAPK signaling pathway in the soleus muscles led to the activation of the ubiquitin‒proteasome system and autophagy, contributing to muscle atrophy. However, treatment with astaxanthin or p38 inhibitors mitigated the muscle structural changes by deactivating the p38 MAPK signaling pathway. These findings provide insight into the role of oxidative stress in muscle alterations in COPD and suggest a potential target for sarcopenia in this context [33].

The effects of cigarette smoke extract (CSE) on macrophage apoptosis and the underlying mechanisms showed that CSE induced apoptosis in macrophages and increased the expression of cleaved caspase 3. Additionally, CSE activated the endoplasmic reticulum stress (ERS) pathway and increased the phosphorylation levels of p38, JNK, and ERK1/2. Importantly, the inhibition of p38 significantly reduced CSE-induced apoptosis, while the inhibition of ERK1/2 promoted apoptosis. Furthermore, CSE increased intracellular calcium levels, and a calcium chelator partially attenuated the apoptosis and phosphorylation of P38 and STAT1 induced by CSE. These findings suggest that CSE induces caspase 3-dependent apoptosis in macrophages through ERS and the intracellular calcium/P38/STAT1 pathway, highlighting the potential role of P38 inhibitors as a therapeutic target in COPD [152].

Additionally, studying the exacerbation in a COPD mouse model by mimicking viral infection (TLR3 ligand) and cigarette smoke (CS) exposure revealed that the TLR3 ligand enhanced the expression of inflammatory cytokines and remodeling factors in pulmonary epithelial cells. However, treatment with p38 inhibitors effectively reduced the expression of inflammatory cytokines and remodeling factors while increasing the expression of E-cadherin. Additionally, p38 inhibitors attenuated airway hyperreactivity induced by the TLR3 ligand. These findings suggest that targeting the TLR3/p38 pathway could be a potential therapeutic approach for managing airway inflammation and remodeling in COPD [153].

To investigate the interaction of inflammation and oxidative stress with protease-antiprotease imbalance, a mouse model was exposed to CSE, which showed an enhanced inflammatory response induced by neutrophil elastase (NE) in bronchial epithelial cells. This enhancement was found to be dependent on the activation of proteinase-activated receptor 2 (PAR2) and the extracellular signal-regulated kinase (ERK) pathway. Interestingly, inhibition of p38 MAPK attenuated the upregulation of PAR2 and the subsequent increase in NE-induced interleukin-8 (IL-8) production [154].

### Insights from clinical trials

Due to the predominant expression of p38α and β proteins in the lungs of COPD patients, therapeutic strategies and clinical trials have primarily focused on these p38 isoforms [15, 31]. There is a negligible chance of cross-interaction for α and β p38 isoforms with their specific inhibitors [155]. Although initial efforts and clinical trials of orally administered p38 inhibitors were made, p38 oral compounds were discontinued for further trials with the emergence of inhaled formulations, except Novartis’ Acumapimod, an oral inhibitor of p38α (IC50 < 1 μM) [156, 157]. A study investigated acumapimod as a part of an ongoing clinical trial (NCT02700919) for the treatment of AECOPD patients (acute exacerbations of COPD). The study showed that short-term therapy with acumapimod could improve lung function indices and decrease inflammation biomarkers, clinical outcomes, and length of hospital stay. Thus, acumapimod was suggested as a potential drug for the treatment of AECOPD, which can reduce the healthcare burden and improve patients’ quality of life [158].

In contrast, losmapimod and dilmapimod, which are oral medications, were withdrawn from phase II clinical trials due to their disappointing results [56, 156, 159]. Similarly, the oral application of losmapimod failed to reduce the frequency of AECOPD [160]. However, a study showed that losmapimod significantly reduced the plasma level of fibrinogen in these patients [149] (Table 4). In contrast, inhaled p38 inhibitors have been suggested to reduce the systemic side effects of COPD medications [161, 162]. Table 4 summarizes the p38 inhibitors that have undergone phase II clinical trials, along with their clinical outcomes. For example, a clinical trial on the effectiveness and safety of inhaled PH-797804 in patients with COPD GOLD stage II/III  [55] showed an improvement in prebronchodilator FEV1 and the transitional dyspnea index (TDI) [55] (Table 4).

**Table 4:** List of clinical trials assessing the efficacy of different p38 inhibitors in COPD

NCT Number^a^	Status	last update	Study Results	Interventions	Dosing	Phases	Enrollment	Publication
NCT02815488	Terminated	March 2019	NA^b^	CHF6297	Single and repeated doses	Phase 2	118	[150]
NCT00642148	Completed	July 27, 2009	NA	GW856553 (Losmapimod)	12-weeks of treatment with GW856553 7.5 mg twice daily	Phase 2	306	[149]
NCT00144859	Completed	July 2005	NA	SB681323 (Dilmapimod)	7.5 mg Per Day	Phase 2	82	NA
NCT02366637	Terminated	May 2015	With Results	PF-03715455 (aka compound 1ab)	Orally inhaled placebo twice a day (BID) for 4 weeks	Phase 2	13	NA
NCT01541852	Completed	February 2014	NA	GW856553 (Losmapimod)	7.5 mg tablet twice daily	Phase 2	72	[163]
NCT01332097	Completed	May 2013	NA	BCT197 (Acumapimod)	single 75 mg oral dose of BCT197 capsules	Phase 2	183	[164]
NCT01218126	Completed	December 21, 2011	With Results	GW856553 (Losmapimod)	three doses of losmapimod (2.5 mg, 7.5 mg and 15 mg) twice daily	Phase 2	604	[56, 159]
NCT02299375	Completed	December 21, 2011	With Results	GW856553 (Losmapimod)	15 mg (mg) twice daily	Phase 2	184	[160]
NCT00559910	Completed	June 30, 2016	NA	PH-797804	0.5, 3, 6 or 10 mg PH-797804 once daily and treated for 6 weeks following a 2-week run-in	Phase 2	230	[55]
NCT01937338	Completed	April 2014	NA	AZD7624	nebulizer solution; 20 mg/mL for inhalation	Phase 1	60	[144]
NCT02238483	Completed	April 4, 2016	With Results	AZD7624	Inhaled AZD7624 solution, 11 mg/mL	Phase 2	213	[144]
NCT01475292	Completed	April 2012	NA	RV568	RV568 50 and 100 ug administered via a nebulizer once daily for 14 days	Phase 2	30	[146]
NCT01703052	Completed	May 2012	NA	CHF6001	a single-dose dry-powder inhaler including 20, 100, 200, 400, 800, 1,600, and 2,000 µg and 2,400, 4,000, and 4,800 µg	Phase 1	74	[165]
NCT02386761	Completed	May 2015	NA	CHF6001	a single-dose dry-powder inhaler including 2,400, 4,000, and 4,800 µg	Phase 1	48	[165]
NCT00439881	Completed	June 2012	NA	SB-681323	single intravenous dose	Phase 1	16	[21]

^a^ClinicalTrials.gov Identifier

^b^*NA* Not available

In an investigation of AZD7624, an inhaled p38α/β inhibitor, for treating COPD patients, it was discovered that while the inhibitor significantly reduced TNF-α and neutrophils in sputum, there was no statistically significant disparity between AZD7624 and placebo among 213 COPD patients concerning the number of days to the first moderate or severe exacerbation or early dropout [144]. Another randomized controlled trial of AZD7624 revealed that although the administration of this inhibitor was safe and well tolerated, it did not offer any clinical advantages in terms of COPD exacerbation management and general lung functions in infrequently exacerbated patients [166] (Table 4).

During a phase II clinical trial, RV-568, also known as JNJ-49095397, a narrow-spectrum inhaled p38 inhibitor, was found to successfully inhibit proinflammatory cytokines in macrophages, airway epithelial cells, and smooth muscle cells [167, 168]. The administration of 100 μg/day RV-568 was well tolerated in COPD patients for seven days [169]. Studies have shown that inhaled RV-568 significantly improves the FEV1 index after 14 days and reduces sputum malondialdehyde and myeloperoxidase levels [170]. Furthermore, another study indicated that RV-568 inhibits the expression of IL-1β and CXCL-8 cytokines by 90% and 73%, respectively, at the highest administered doses [171]. Moreover, other studies have shown the anti-inflammatory properties and lung function benefits of RV-568, which are synergistically augmented by corticosteroid administration [146] (Table 4).

In a clinical study of patients with GOLD II/III COPD, the oral p38 inhibitor PH-797804 was found to reduce the levels of CRP, a systemic inflammatory biomarker, and significantly improve lung function after 6 weeks of administration. However, there was no significant difference in the levels of CC16, IL-6, surfactant protein D (SPD), or fibrinogen compared to placebo controls [55] (Table 3 and Fig. 3). Another oral p38 inhibitor, SB-681323, has been shown to decrease TNF-α levels in LPS-stimulated blood samples of COPD patients, while prednisolone had no effect on TNF-α levels in these patients [44] (Table 3 and Fig. 3). A transcriptomic study of blood inflammatory factors in COPD patients found that a single dose of oral SB-681323 downregulated several inflammatory factors associated with p38 inhibition by mediating the involvement of IL-1β and STAT1 in [54].

Clinical trials of p38 MAPK inhibitors in humans have shown that these inhibitors decrease serum levels of both TNF-α and IL-6 after LPS administration, as well as acute phase reactants associated with inflammation, such as serum erythrocyte sedimentation rate (ESR) and c-reactive protein (CRP) [145].

A recently published systematic review and meta-analysis evaluated the safety and efficacy of p38 MAPKIs as an alternative to corticosteroids in the treatment of COPD in ten randomized controlled trials (RCTs) involving a total of 1,751 participants [44, 55, 56, 144, 146, 149, 158, 160, 172, 173]. The analysis focused on various outcome measures, including lung function, inflammatory biomarkers, and quality of life [174]. The results indicated that while p38 MAPKIs were found to be safe, they did not demonstrate significant effects compared to placebo in terms of efficacy. In summary, while the present systematic review and meta-analysis suggest that p38 MAPKIs do not demonstrate significant efficacy compared to placebo in COPD treatment, it is imperative to exercise caution when interpreting these findings due to the limited number of included studies and the heterogeneity resulting from combining different p38 MAPKIs. Further research is warranted to provide a more comprehensive understanding of the role and potential benefits of p38 MAPKIs in the management of COPD.

## Conclusion

In the context of COPD, p38 inhibition has been observed to effectively mitigate inflammation and oxidative stress in the pulmonary and muscular tissues of affected individuals, which are closely associated with airflow restriction, respiratory distress, and reduced physical tolerance [55, 174]. Moreover, the p38 inhibitors have demonstrated the capability to improve key lung function parameters and alleviate dyspnea in COPD patients, as assessed through measurements such as forced expiratory volume in 1 s (FEV1), forced vital capacity (FVC), and transition dyspnea index (TDI) [175]. Notably, p38 inhibitors appear to offer superior therapeutic outcomes compared to steroids for specific subsets of COPD patients, given that steroids possess limited anti-inflammatory efficacy and are not recommended as standalone treatments for COPD [55, 174]. However, it is important to acknowledge that p38 inhibitors may entail undesirable adverse effects, including liver toxicity and cutaneous reactions, which impose restrictions on their clinical usage and raise concerns regarding their safety [16]. Additionally, the transient efficacy of p38 inhibitors, which tends to dissipate within a matter of weeks, can be attributed to the intricate and redundant nature of the inflammatory network. Inhibition of one pathway may inadvertently activate alternative pathways that perpetuate inflammation. Furthermore, the effects of p38 inhibitors can vary among different subgroups of COPD patients, contingent upon their specific inflammatory phenotypes and the severity of their disease. The long-term implications of p38 inhibitors on COPD progression and mortality remain uncertain since most clinical trials have focused primarily on short-term assessments of lung function and symptomatology [16, 174].

Additionally, activation of p38 in distinct cell types, including macrophages and neutrophils, can induce inflammatory responses and tissue damage, thereby contributing to the pathogenesis of COPD. For example, p38 activation in these inflammatory cells can elicit the release of pro-inflammatory cytokines and chemokines, perpetuating the chronic inflammation observed in COPD. In such cases, inhibiting p38 signaling holds potential for reducing excessive inflammation and attenuating disease progression. However, it is imperative to acknowledge that the p38 signaling pathway also assumes indispensable roles in cellular processes that are fundamental for maintaining tissue homeostasis and facilitating repair mechanisms. For instance, activation of p38 in epithelial cells has been shown to enhance cell survival, preserve the integrity of the epithelial barrier, and facilitate tissue repair mechanisms. Consequently, inhibiting p38 signaling in these cells has the potential to disrupt these advantageous activities and compromise the regenerative capacity of lung tissue. Therefore, while the inhibition of p38 signaling may manifest anti-inflammatory effects, it is crucial to consider the potential ramifications on other cell functions and the delicate equilibrium between inflammation and tissue repair in the context of COPD. The challenge lies in selectively targeting the detrimental aspects of p38 signaling without compromising the beneficial functions, necessitating further research and a more comprehensive understanding of the intricate interplay between diverse cell types and signaling pathways implicated in COPD pathogenesis [113].

Finally, it is crucial to recognize that COPD is a multifaceted and intricate disease with a plethora of underlying mechanisms that contribute to its onset and progression. While inflammation, oxidative stress, and imbalances in tissue repair are pivotal factors in the pathogenesis of COPD, it is imperative to acknowledge the involvement of other elements as well, including genetic predisposition, environmental exposures, and immune dysregulation. COPD should not be regarded solely as an inflammatory condition; however, inflammation undeniably plays a significant role in its development and advancement. Persistent inflammation in the airways and lung tissue is a defining characteristic of COPD and contributes to the manifestation of symptoms and complications. Nonetheless, it is vital to note that COPD encompasses a complex interplay of multiple underlying mechanisms beyond inflammation. In addition to inflammation, COPD involves oxidative stress, imbalances in protease-antiprotease activity, impaired tissue repair processes, and structural alterations in the lungs, among other factors. Consequently, focusing solely on inhibiting inflammation may not comprehensively address all facets of the disease and could potentially lead to a misleading perspective.

As stated, the impaired clearance of senescent cells in the lungs of COPD patients, including senescent epithelial cells and inflammatory cells such as macrophages and neutrophils, can lead to their accumulation. This accumulation poses a challenge in COPD, as these senescent cells contribute to chronic inflammation and tissue damage. Dysregulated signaling pathways, including the activation of p38, play a crucial role in driving inflammation and worsening the disease. Therefore, targeting p38 signaling has emerged as a potential therapeutic approach for COPD. Inhibiting p38 activation can help reduce the release of inflammatory mediators and mitigate the inflammatory cascade, leading to a reduction in tissue damage and improvement in lung function. However, it is important to exercise caution when inhibiting p38, as it also plays critical roles in essential cellular processes required for tissue homeostasis and repair. Striking a balance between suppressing inflammation and preserving beneficial cellular functions is crucial when considering p38 as a therapeutic target in COPD management.

By unraveling the intricate mechanisms at play, we can develop more targeted and effective therapies that selectively modulate p38 activity, striking a balance between suppressing inflammation and preserving beneficial cellular functions for optimal COPD management. To effectively manage COPD, treatment strategies should be aimed at restoring the equilibrium of tissue repair mechanisms, alleviating oxidative stress, and targeting specific molecular pathways implicated in COPD progression. The utilization of combinatorial therapies, involving the concurrent use of multiple therapeutic agents targeting distinct aspects of the disease, is gaining prominence in COPD management. This approach recognizes the multifaceted nature of COPD and strives to address the diverse mechanisms contributing to disease progression. By concurrently targeting repair imbalances, reducing inflammation, and addressing other factors, combinatorial therapies have the potential to offer more comprehensive and effective treatment outcomes. It is important to note that the development and implementation of combinatorial therapies necessitate extensive research, clinical trials, and personalized approaches to tailor treatment strategies to individual patients. The ultimate objective is to achieve a delicate balance between suppressing inflammation and promoting tissue repair and homeostasis to optimize outcomes for individuals with COPD.

## Data Availability

Not applicable.

## Abbreviations

Ams: Alveolar macrophages
ARG1: Arginase 1
ASK1: Apoptosis signal-regulating kinase 1
BAL: Bronchoalveolar lavage
CCL5: Chemokine (C-C motif) ligand 5
COPD: Chronic obstructive pulmonary disease
COX-2: Cyclooxygenase-2 expression
CSE: Cigarette smoke extract
DUSPs: Dual-specificity phosphatases
ECM: Extracellular matrix
EndMT: Endothelial-to-mesenchymal transition
EPAS1: Domain-containing protein 1
ERS: Endoplasmic reticulum stress
FEV1: Forced expiratory volume in 1 s
FVC: Forced vital capacity
GM-CSF: Granulocyte-macrophage colony-stimulating factor
HASMC: Human airway smooth muscle cells
HCKs: Hematopoietic cell kinase
HSP27: Heat Shock Protein 27
IFN-γ: Interferon gamma
IL-1β: Interleukin 1 beta
IL-6: Interleukin 6
IL-8: Interleukin 8
IMs: Interstitial macrophages
LABAs: Long-acting β2-agonists
LAMAs: Long-acting muscarinic antagonists
LPS: Lipopolysaccharide
MAP3K1: Mitogen-activated protein kinase kinase kinase 1
MKPs: MAP kinase phosphatases
MLK3: Mixed lineage kinase 3
Mo-AMs: Monocyte-derived alveolar macrophages
mRNA: Messenger RNA
MSCs: Mesenchymal stem cells
PBMCs: Peripheral blood mononuclear cells
PGE2: Prostaglandin E2
PP2A/C: Protein phosphatase 2A/C
RANTES: Regulated on activation, normal T-cell expressed and secreted
ROS: Reactive oxygen species
SMCs: Smooth muscle cells
SPD: Surfactant protein D
SRC: Steroid Receptor Coactivator
TAK1: TGF-β-activated kinase 1
TDI: Transition dyspnea index
TGF-β: Transforming growth factor β
TLR4: Toll-like receptor 4
TNF-α: Tumor necrosis factor-alpha
TR-AMs: Tissue-resident alveolar macrophages
VEGF: Vascular endothelial growth factor
WHO: World Health Organization
Wip1: Wild-type p53-induced phosphatase 1
